# Bile accelerates carcinogenic processes in pancreatic ductal adenocarcinoma cells through the overexpression of MUC4

**DOI:** 10.1038/s41598-020-79181-6

**Published:** 2020-12-16

**Authors:** Eleonóra Gál, Zoltán Veréb, Lajos Kemény, Dávid Rakk, András Szekeres, Eszter Becskeházi, László Tiszlavicz, Tamás Takács, László Czakó, Péter Hegyi, Viktória Venglovecz

**Affiliations:** 1 Department of Pharmacology and Pharmacotherapy, University of Szeged, 6720 Szeged, Hungary; 2 Regenerative Medicine and Cellular Pharmacology Research Laboratory, Department of Dermatology and Allergology, University of Szeged, Szeged, Hungary; 3 HCEMM SZTE Skin Research Group, University of Szeged, Szeged, Hungary; 4 Department of Microbiology, University of Szeged, Szeged, Hungary; 5 Department of Pathology, University of Szeged, Szeged, Hungary; 6 First Department of Medicine, University of Szeged, Szeged, Hungary; 7 Institute for Translational Medicine, Medical School, Szentágothai Research Centre, University of Pécs, Pécs, Hungary; 8 Division of Gastroenterology, First Department of Medicine, Medical School, University of Pécs, Pécs, Hungary

**Keywords:** Gastrointestinal cancer, Cancer, Molecular biology, Gastroenterology

## Abstract

Pancreatic cancer (PC) is one of the leading causes of mortality rate globally and is usually associated with obstructive jaundice (OJ). Up to date, there is no clear consensus on whether biliary decompression should be performed prior to surgery and how high levels of serum bile affects the outcome of PC. Therefore, our study aims were to characterise the effect of bile acids (BAs) on carcinogenic processes using pancreatic ductal adenocarcinoma (PDAC) cell lines and to investigate the underlying mechanisms. Liquid chromatography-mass spectrometry was used to determine the serum concentrations of BAs. The effects of BAs on tumour progression were investigated using different assays. Mucin expressions were studied in normal and PDAC cell lines and in human samples at gene and protein levels and results were validated with gene silencing. The levels of BAs were significantly higher in the PDAC + OJ group compared to the healthy control. Treating PDAC cells with different BAs or with human serum obtained from PDAC + OJ patients enhanced the rate of proliferation, migration, adhesion, colony forming, and the expression of MUC4. In PDAC + OJ patients, MUC4 expression was higher and the 4-year survival rate was lower compare to PDAC patients. Silencing of MUC4 decreased BAs-induced carcinogenic processes in PDAC cells. Our results show that BAs promote carcinogenic process in PDAC cells, in which the increased expression of MUC4 plays an important role. Based on these results, we assume that in PC patients, where the disease is associated with OJ, the early treatment of biliary obstruction improves life expectancy.

## Introduction

Pancreatic cancer (PC) is associated with extremely poor survival and high mortality rate. Currently, PC is the seventh leading cause of cancer-related deaths worldwide^1^. One of the most common reasons for the poor clinical outcome is the lack of specific symptoms; as a result, approximately 80% of patients are diagnosed at an advanced stage, when most of them are inoperable^2–5^. The most common form of PC is pancreatic ductal adenocarcinoma (PDAC), which is responsible for approximately 90% of cases^6^. Most of the PDAC arises from ductal cells in the head of the pancreas. As tumour grows, it prevents the flow of bile and, as a result, obstructive jaundice (OJ) develops. Elevated serum levels of bile acids (BAs) influence the function of several organs; they have also proved to have tumorigenic potential in both gastrointestinal and breast cancer^7^. Although surgical intervention is widely regarded as the most effective way for the treatment of PC^3,8^, the use of preoperative biliary stenting is often the basis for debate and it usually takes time to make a decision^9–11^. Moreover, there is no consensus regarding the role of BAs in the initiation and progression of PC^12^. Some studies indicate that BAs inhibit the proliferation of PC cells due to their cytotoxic properties^13,14^, while others found that BAs promote tumour development and progression by increasing the expression of COX-2 or mucins^15–17^.

In recent years, considerable attention has been paid to the diagnostic use of mucins in PC. Twenty-one mucin genes have been identified in humans and, among them, MUC1, -4 and -5AC proved to be potential biomarkers to assess the progression of PC. These genes are mainly overexpressed in PC, play role in tumour cell growth and associate with a poor outcome for PC patients^16,18–20^. Several studies indicate that BAs play an extensive role in tumour progression by altering the expression of mucins^17,21–25^. In the oesophagus, BAs upregulate mucin expression, in which phosphatidylinositol 3-kinase and nuclear factor-κB (NK-κB) signalling pathways play a role^17,23,24^. The role of NK-κB in bile-induced mucin expression has also been implicated in gastric epithelial cells^25^. In contrast, there has not been in-depth study pertaining to the pancreas; thus, this study aims (i) to investigate how BAs treatment affect the behaviour of PDCA cells and ii) to identify the mechanisms that mediate the effects of BAs.

We have shown that BAs increase the tumorigenic potential of PDAC cells, through the overexpression of MUC4. In addition, we investigated the expression of MUC4 in human PC samples and identified a relation between the presence of OJ and increased expression of MUC4. Moreover, we have found that the 4-year overall survival rate of the PDAC + OJ patients was significantly poorer than that of the PDAC patients. Taken together our results show that bile accelerates carcinogenic processes, which can be of great importance in the therapy of PC.

## Materials and methods

### Ethical aspects

The clinical part of the study was carried out with the approval of the Ethics Committee of the University of Szeged (No.: 4714), followed by the EU Member States' Directive 2004/23/EC on presumed consent practice for tissue collection, the guidelines of the Helsinki Declaration and GDRP. Written informed consent was obtained from all patients and healthy volunteers for sample and data collection.

### Pathological characterisation of the patients

Serum levels of BAs were investigated in PDAC patients with OJ (average age: 72.6 ± 9.8; male/female ratio: 5/5) or without OJ (average age: 80 ± 2.5; male/female ratio: 2/3) and in healthy volunteers (average age: 40.9 ± 18.77; male/female ratio: 6/8). In all groups, BAs were identified in fasting serum samples. See Table 1 for the details of patients.

**Table 1:** Clinicopathological characteristics of pancreatic cancer patients selected for serum bile acid measurements.

Variable	PDAC + OJ (n = 10)		PDAC (n = 5)			NORMAL (n = 14)		
	n	(%)	n	(%)	*p value*	n	(%)	*p value*
**Gender**								
Male	5	(50.0)	2	(40.0)		6	(42.9)	
Female	5	(50.0)	3	(60.0)	0.7376	8	(57.1)	0.9930
**Age**								
˂65	2	(20.0)	0	(0.0)		10	(71.0)	
≥ 65	8	(80.0)	5	(100.0)	0.5877	4	(29.0)	0.7125
**Location of primary tumor**								
Papilla of Vater	3	(30.0)	0	(0.0)				
Head	4	(40.0)	5	(100.0)				
Head/Body	2	(20.0)	0	(0.0)				
Body	1	(10.0)	0	(0.0)				
Tail	0	(0.0)	0	(0.0)	0.4369			
**Hystological type**								
Well differentiated	0	(0.0)	0	(0.0)				
Moderately differentiated	7	(70.0)	5	(100.0)				
Poorly differentiated	3	(30.0)	0	(0.0)	0.5599			
**Stage of the cancer**								
II	0	(0.0)	0	(0.0)				
III	4	(40.0)	4	(80.0)				
IV	6	(60.0)	1	(20.0)	0.4456			
**Lymphatic invasion**								
Negative	7	(70.0)	5	(100.0)				
Positive	3	(30.0)	0	(0.0)	0.5166			
**Metastasis**								
Lung	0	(0.0)	0	(0.0)				
Liver	4	(40.0)	3	(60.0)				
Colon	1	(10.0)	2	(30.0)				
Gall bladder	0	(0.0)	0	(0.0)	0.6540			
**Bile acids in human serum (ng/ml)**								
GCA	9890.5 ± 3267.1	(27.43)	41.46 ± 53.45	(5.65)		27.35 ± 26.66	(6.82)	
GDCA	217.22 ± 193.27	(0.62)	67.83 ± 93.28	(9.25)		70.01 ± 86.59	(17.44)	
GCDCA	4770.8 ± 2375.1	(13.23)	339.93 ± 376.6	(46.31)		186.77 ± 108.37	(46.54)	
TCA	13,299 ± 2827.1	(36.88)	98.96 ± 57.09	(13.49)		0.0 ±	(0.0)	
TDCA	0.0 ±	(0.0)	47.6 ± 27.4847.6 ± 27.48	(6.48)		61.32 ± 53.02	(15.28)	
TCDCA	7878.1 ± 1644.3	(21.84)	138.07 ± 126.27	(18.81)		55.85 ± 31.55	(13.92)	
Total serum bile acids	36,055.7 ± 2182.2		733.85 ± 118.7		0.0275	401.3 ± 35.38		0.0228

We performed immunohistochemistry on pancreatic samples obtained from 65 patients. These patients have been classified into the following groups: (1) PDAC (average age: 65.6 ± 1.4; male/female ratio: 17/8), (2) PDAC + OJ (average age: 66.4 ± 1.7; male/female ratio: 13/12), (3) neuroendocrine tumour (NE) (average age: 68.1 ± 2.25; male/female ratio: 8/3) and (4) control group (average age: 62.75 ± 3.3; male/female ratio: 1/3). All the samples were obtained from surgical resection or biopsy. Pathological characterisation of the PDAC tumours, including PDAC + OJ tumours, confirmed that most of them were moderately differentiated (grade 2, n = 30); 14 tumours were poorly differentiated (grade 3), whereas 6 tumours were well-differentiated (grade 1). The majority of PDAC developed in the head. Among the PDAC patients, 22 were in stage IV, 22 in stage III and 6 in stage II. Metastasis was present in 25 cases. We followed up on all patients for 48 months, during which time all PDAC and PDAC + OJ patients died. See Table 2 for the details of patients.

**Table 2:** Clinicopathological characteristics of pancreatic cancer patients selected for MUC4 staining.

Variable	PDAC + OJ (n = 25)		PDAC (n = 25)		NE (n = 11)		NORMAL (n = 4)	
	n	(%)	n	(%)	n	(%)	n	(%)
**Gender**								
Male	13	(52.0)	17	(68.0)	8	(72.7)	1	(25.0)
Female	12	(48.0)	8	(32.0)	3	(27.3)	3	(75.0)
**Age**								
˂ 65	6	(24.0)	10	(40.0)	4	(36.36)	4	(100.0)
≥ 65	19	(76.0)	15	(60.0)	7	(63.64)	0	(0.0)
**Location of primary tumor**								
Papilla of Vater	3	(12.0)	0	(0.0)	0	(0.0)		
Head	22	(88.0)	18	(72.0)	6	(54.54)		
Head/Body	0	(0.0)	5	(20.0)	0	(0.0)		
Body	0	(0.0)	1	(4.0)	1	(9.1)		
Tail	0	(0.0)	1	(4.0)	4	(36.36)		
**Hystological type**								
Well differentiated	3	(12.0)	3	(12.0)	4	(36.37)		
Moderately differentiated	12	(48.0)	18	(72.0)	7	(63.63)		
Poorly differentiated	10	(40.0)	4	(16.0)	0	(0.0)		
**Stage of the cancer**								
II	2	(8.0)	4	(16.0)	8	(72.73)		
III	10	(40.0)	12	(48.0)	3	(27.27)		
IV	13	(52.0)	9	36.0)				
**Lymphatic invasion**								
Negative	17	(68.0)	20	(80.0)	11	(100.0)		
Positive	8	(32.0)	5	(20.0)	0	(0.0)		
**Metastasis**								
Lung	2	(8.0)	4	(16.0)	0	(0.0)		
Liver	6	(24.0)	10	(40.0)	2	(18.18)		
Colon	0	(0.0)	2	(8.0)	0	(0.0)		
Gall bladder	1	(4.0)	0	(0.0)	0	(0.0)		

### Chemicals and solutions

TaqMan gene expression assays, MTT 3-(4,5-dimethylthianol-2-yl)-2,5-diphenyltetrazolium bromide (MTT), siRNAs for MUC4 (Cat.No.:AM 16708), and the oligofectamine transfection kit were obtained from Thermo Fisher Scientific (Watham, MA,USA). Mouse MUC4 monoclonal IgG1 antibody was ordered from Santa Cruz Biotechnology (Cat.No.:sc-33654; Dallas, TX, USA). Guinea pig vimentin polyclonal antibody was ordered from Fitzgerald Industries International (Cat.No.:20R-VP044, Acton, MA, USA). Texas Red-AffiniPure Goat anti Guinea Pig IgG secondary antibody was from Jackson ImmunoResearch Laboratories, Inc. (Cat.No.:106-075-003; Cambridgeshire, UK). Technical Manual Cell Counting Kit-8 (CCK) was obtained from Dojindo Molecular Technologies (Rockville, MD, USA). Glycodeoxycholic acid (GDCA) was from Cayman Chemical Company (Michigan, MI, USA). BAs (glycocholic acid (GCA); taurocholic acid (TCA); taurodeoxycholic acid (TDCA); glycochenodeoxycholic acid (GCDCA); taurochenodeoxycholic acid (TCDCA)) and all other laboratory chemicals were ordered from Sigma-Aldrich Kft. (Budapest, Hungary). For more details on gene expression assays see Suppl. Table S1.

### Cell lines and culture conditions

The human PDAC cell lines, Capan-1, Miapaca-2, Panc-1 and BxPC-3 were obtained from American Type Culture Collection (Manassas, VA, USA). Capan-1 and BxPC-3 cells were cultured in RPMI-1640 media supplemented with 15% (v/v) fetal bovine serum (FBS); 1% (v/v) L-glutamine and 2% (v/v) antimycoticum/antibioticum. Miapaca-2 and Panc-1 were maintained in Dulbecco’s Modified Eagle’s Medium high glucose medium supplemented with 10% (v/v) FBS, 1% (v/v) l-glutamin, 2.5% (v/v) horse serum, and 1% (v/v) pencillin/streptomycin. HPDEC, human pancreatic ductal epithelial cell line was ordered from Hölzel Diagnostika Handels GMBH (Köln, Germany) and the cells were cultured in keratinocyte serum-free media supplied with prequalified human recombinant Epidermal Growth Factor and Bovine Pituitary Extract. The cells were cultured under standard conditions (37 °C and 5% CO_2_) the medium was replaced in every alternate day and the cells were cultured at 100% confluence. Capan-1 cells were used between 30 and 35, BxPC-3 cells between 2 and 5, Panc-1 and Miapaca-2 cells between 20 and 25 and HPDEC cells between 9 and 11 passage numbers.

### Bile acid treatment

The cells were seeded into 25 cm^2^ tissue culture flasks or 96-well tissue plates, two days before the BAs treatment. The treatment was performed with six different types of BAs (GCA, TCA, GDCA, TDCA, GCDCA, TCDCA) in two different concentrations (100 and 500 µM), for 24, 48 and 72 h. Bile acid cocktail (BAC) contained all BAs in equal concentrations, with a final concentration of 500 µM.

### Cell adhesion assay

We coated 96-well tissue plates with 40 µg/ml type 1 collagen from rat-tail in PBS at 4 °C. Next, we added 100 µl of cell suspension (10^5^ cells/ml) to each of the coated wells and incubated the cells at 37 °C for 20 min to allow them to adhere to the surface. After washing, the cells were incubated with BAs and 10 µl of MTT substrate was added to each well. MTT-treated cells were then lysed in DMSO and absorbance was measured using a FLUOstar OPTIMA Spectrophotometer (BMG Labtech, Ortenberg, Germany) at 560 nm with background subtraction at 620 nm.

### Proliferation

For proliferation, 100 µl of cell suspension was seeded into a 96-well plate (5 × 10^3^ cells/well), then the cells were incubated with BAs. After the treatments, 10 µl of CCK8 solution was added to each well and the cells were incubated for further 3 h. We measured absorbance at 450 nm using a FLUOstar OPTIMA Spectrophotometer (BMG Labtech, Ortenberg, Germany).

### Cytotoxicity assay

For cytotoxicity assay, 100 µl of cell suspension was seeded into a 96-well plate (2 × 10^4^ cells/well) and allowed to adhere overnight. On the following day, the cells were incubated with BAs then 100 µl supernatant from each of the wells was carefully transferred into a new 96-well plate containing 100 µl reaction mixture. We then measured lactate dehydrogenase (LDH) activity at 490 nm using a FLUOstar OPTIMA Spectrophotometer (BMG Labtech, Ortenberg, Germany). For background controls, we measured 200 µl assay medium, without cells. For low controls, we used 100 µl cell suspension and 100 µl assay medium. In the case of high controls, the mixture of 100 µl cell suspension and 100 µl Triton-X 100 (0.1%) solution was measured. The LDH release induced by Triton-X 100 was assigned to 100%. The average absorbance values of each of the triplicates were calculated and the average value of the background control (LDH activity contained in the assay medium) was subtracted from each of the samples to reduce background noises. We then calculated the percentage of cytotoxicity using the following formula: Cytotoxicity (%) = (exp. value–low control/high control-low control)*100. Low control determines the LDH activity released from the untreated normal cells (spontaneous LDH release), whereas high control determines the maximum releasable LDH activity in the cells (maximum LDH release).

### Wound healing assay

Cells were seeded onto 24-well cell culture plates in a 2.5 × 10^5^ cell density and allowed to adhere overnight. On the following day, the confluent monolayer was gently scratched using P2 tips. Only the wells containing even-sided and sharp-edged wounds were used for experiments. After gentle washing with the complete medium, we added BAs to the wells. We carried out automated time lapse imaging using an Olympus IX83 inverted microscope with Olympus ScanR screening platform (Olympus, Japan) upgraded with OKOLAB incubator system (with gas, temperature, and humidity controller; Pozzuoli, NA, Italy). Digital images were analysed by Image J.

### Clonogenic assay

Capan-1 and BxPC-3 cells (10^3^ cells/well) were seeded onto 6-well cell culture plates and allowed to adhere overnight. On the following day, the cells were treated with BAs then the normal media was given back. The cells were allowed to grow until day 9 after which the media was removed, and the cells were washed with PBS, fixed with methanol-ethanol solutions (3:1 dilution) and then stained with Giemsa. Olympus IX83 microscope-based screening platform was used for image acquisition and the Olympus Cellsense Dimension software was used for automated object detection, classification and measurement to enumerate colonies organised by the treated and untreated cells.

### Invasion assay

For the invasion assay Matrigel-coated transwell inserts were used. 200 µl cells (~ 2.5 × 10^5^/ml in serum-free medium) were added into the inserts whereas the lower chambers contained 750 µl complete medium with or without BAs. Cells were than incubated at 37 °C for 24–72 h in 5% CO_2_ in a humidified incubator. Cells that migrated to the bottom surface were fixed in formaldehyde (3.7% in PBS) for 5 min, permeabilized with 100% methanol and stained with Giemsa dye for 30 min. The non-invading cells on the upper surface of the membrane were gently scraped off using a cotton swab. Invasion was quantified by counting the average number of invaded cells in five different microscopic fields in each treatment. Percent invasion was calculated from the mean of the average number of invaded cells obtained from 3 independent experiments.

### siRNA silencing

MUC4 expression was silenced transiently, using MUC4-targeted siRNA oligonucleotides. Transfection was performed with Oligofectamine™ Transfection Reagent following the manufacturer’s instructions. We then plated 2 × 10^5^ cells per well onto 6-well plates a day before the transfection. At 50–60% confluency, the MUC4-targeted siRNAs were transfected and the cells were incubated for 72 h. MUC4 mRNA and protein levels were assessed by RT-PCR and immunocytochemistry, respectively.

### RT-PCR

The total RNA was isolated from the cells using the NucleoSpin RNA Kit (Macherey–Nagel, Düren, Germany). Two micrograms of RNA were reverse-transcripted using the High-Capacity cDNA Reverse Transcription Kit (Applied Biosystems, Foster City, USA). Real-time PCR reactions of samples were performed with TaqMan RT-PCR assays (Supplementary Table S1) from Thermo Fisher Scientific (Darmstadt, Germany). Reactions were carried out with ABI PRISM 7000 Sequence Detection System (Applied Biosystems, Foster City, CA, USA) platform with the following conditions: 10 min initial denaturation at 95 °C, followed by 40 steps cycles: 15 s at 95 °C and 1 min at 60 °C. Fluorescein dye (FAM) intensity was detected after each cycle. All the samples were run in triplicates and non-template control sample was used for each PCR run to check the primer-dimer formation. The expression level of the gene of interest was normalised to the human β-actin (*Actb*) housekeeping gene (ΔCT), and then relative gene expression ratios were calculated using the ΔΔC_T_ method as previously described^26,27^. The results were expressed as fold changes (2^−ΔΔCT^). Genes with expression values less than or equal to 0.5 were considered to be down-regulated, whereas the values higher than or equal to 2 were considered to be upregulated. Values ranging from 0.51 to 1.99 were not considered to be significant.

### Immunostainings

Immunocytochemistry (ICC) was performed using cytospin preparation during which 100 µl (2 × 10^6^ cells/ml) of cell suspension was added to 100 µl of neutral formalin buffer and incubated for 5 min. After the incubation, 100 µl from this mixture were spin (Shandon Cytospin3, Marshall Scientific, Cambridge, MA, US) to an Ultra Plus Microscope Slide (Thermo Fisher Scientific, Darmstadt, Germany). Pre-treatment was carried out with heat-induced epitope retrieval procedure using PT Link (Autostainer Link 48, Agilent, Dako, Santa Clara, CA, US) with EnVision ™ Flex Target Retrieval Solution for 20 min at 92 °C in low pH (pH 6,1; citrate buffer). Slides were then washed with EnVision™ Wash Buffer (20 ×) for 5 min. The endogenous peroxidase blocking was carried out with EnVision™ Flex Peroxidase Blocking Reagent. For staining procedure, the slides were incubated with MUC4 (1:100 dilutions) primary antibodies for 30 min. After incubation, the slides were washed and incubated with secondary antibody (EnVision™ Flex/HPR anti-mouse/rabbit) for 30 min. For visualisation, the Ultra View Universal diaminobenzidine (DAB) Detection Kit (EnVision™ Flex DAB) was applied and nuclear staining was carried out with EnVision™ Flex Hematoxylin. After the staining procedure, the slides were mounted with Xylene Substitute mount (Shandon, Marshall Scientific, Cambridge, MA, US). All specimens were scanned by the Olympus IX83-based system and the pictures were further analysed by ImageJ, whereas the intensities of the pixels of the DAB staining were quantified. In the case of vimentin staining, Capan-1 cells (20,000/chamber) were seeded on chamber slides, fixed with 3.6% paraformaldehyde and permeabilized with 0.2% Triton X-100 and 0.3% protease-free bovine serum albumin. Cells were then incubated with 10% donkey serum to reduce non-specific binding than anti-vimentin primary antibody (1:100 dilution) was added to the chambers and slides were incubated overnight in moist atmosphere at 4 °C. Chamber slides were then washed with PBS and incubated with TexasRed-conjugated anti-mouse secondary antibody (1:400 dilution) for 60 min at RT. Nuclei were counterstained with Hoechst 33342. Slides were then mounted and observed by a Fluowiew 10i-W confocal microscopy (Olympus, Budapest, Hungary). In the human pancreatic samples, MUC4 expression was analysed using formalin-fixed, and paraffin-embedded tissue specimens were obtained from patients. Control tissues (n = 4) were collected from the tumour-free region of the pancreas of patients with NE tumour. Briefly, 3 to 4 µm thick sections of section specimens were deparaffinised in xylene and rehydrated in graded ethanol. The diagnosis was assessed by a pathologist after staining the sections with haematoxylin–eosin–saffron. Immunohistochemistry (IHC) was performed as described above, but the slices were incubated with the primary MUC4 antibody for 60 min. Quantification of MUC4 expression was evaluated using the method described by Rachagani et al.^28^.

### LC–MS measurement of the serum samples

The measurement of BAs was based on the work of Ghaffarzadegan et al. with slight modifications^29^. The stock solution contained 1 mg/ml of each BA in methanol was used to form a seven-point calibration curve ranging from 5–1000 ng/ml determining the concentration of BA in serum samples. In the calibration solutions the concentration of internal standard (IS) were set to 700 ng/ml and 210 ng/ml for GCDA-D4 (used for TCA, GCA, GCDA and GDCA) and DCA-D4 (used for TDCA and TCDCA), respectively. The frozen serum samples were allowed to thaw at 25 °C, then 50 µl of each was spiked with 175 µl methanol, containing IS in 400 ng/ml (GCDA-D4) and 120 ng/ml (DCA-D4). Samples were vortexed (LSE W, Corning, USA) for 1 min then incubated for 30 min in -20 °C. The incubated samples were centrifuged (Heraus Fresco 17, Thermo Scientific, USA) at 13,000 rpm for 15 min in 10 °C. The supernatant was transferred to microcentrifuge tubes and evaporated with vacuum concentrator (Savant SC250EXP, Thermo Scientific, USA) at 40 °C and 1 mbar for 60 min. The residue was dissolved in 100 µl water and 5 µl was subjected to a Nexera XR UHPLC system (Shimadzu, Japan) coupled with a TSQ Quantum Access mass spectrometer (Thermo Scientific, USA). The separations were performed on a Purospher Star RP-18 Hibar HR 100 × 2.1 mm, 2 µm reversed phase column (Merck KGaA, Germany) tempered at 50 °C. The solvent A was 0.1% formic acid in water and the solvent B was 0.1% formic acid in methanol:acetonitrile 1:1. The flow rate was 300 µl/min and the following gradient elution was applied: 0–0.5 min, 30% B; at 1.5 min, 50% B; at 9 min, 58% B; at 9.5 min 85% B; at 12 min, 95% B; at 15 min, 95% B; at 15.5 min, 30% B; at 20 min, 30% B. The mass spectrometer operated in negative ionization mode using HESI ion source where the spray voltage was 3500 V, the vaporizer temperature was 350 °C, the ion-transfer capillary temperature was 275 °C, the sheath gas pressure was 30 in arbitrary units and the aux gas pressure was 20 in arbitrary units. The BAs were detected in SRM mode using two mass transitions for each analyte at the following retention times: TCA (5.3 min, *m/z* 514.3 → 124.7/60.0), GCA (6.7 min, m/z 464.3 → 402.3/75.60), TCDCA (7. 2 min, m/z 498.0 → 125.2/108.3), TDCA (7.9 min, m/z 497.9 → 125.2/108.3), GCDA (8.8 min, m/z 448.2 → 75.6/330.5), GDCA (9.6 min, m/z 448.3 → 402.3/75.6), DCA-D4 (11.9 min, m/z 395.4 → 349.8/330.5) and GCDA-D4 (8.8 min, m/z 452.3 → 390.4/387.6). For the instrument control and the data evaluations, the TraceFinder 4.1 software (Thermo Scientific, USA) was applied.

### Statistical analysis

Quantitative variables were described as means ± SE. Significant differences between groups were performed by ANOVA, p ≤ 0.05 were accepted as significant. Survival curves were prepared using the method of Kaplan and Meier, and differences in survival were studied by the Log-rank test.

## Results

### Serum levels of bile acids in PDAC patients

**Figure 1:**
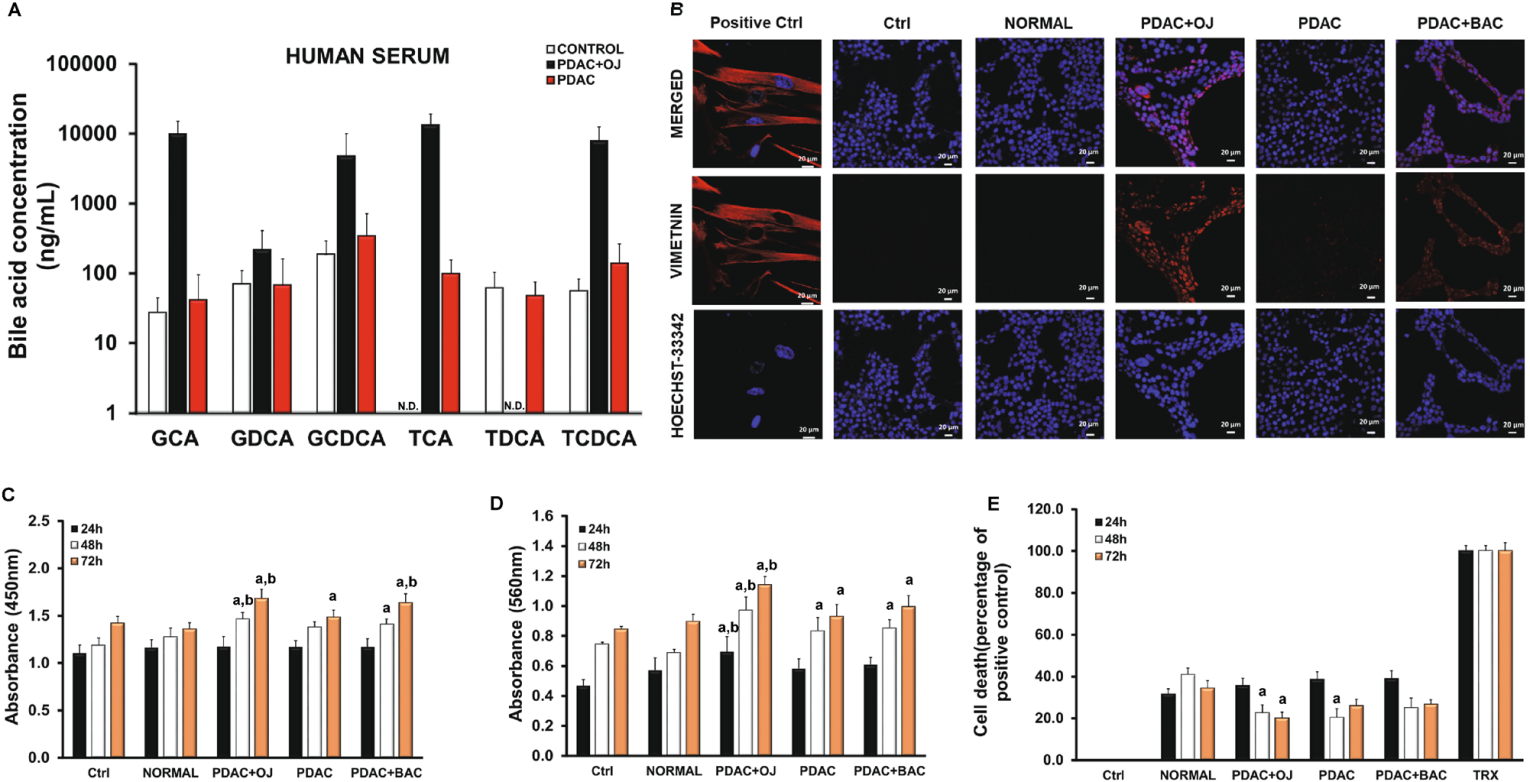
Serum levels of bile acids in pancreatic cancer patients and the effect of serum on Capan-1 cells. (**A**) Serum levels of bile acids (BAs) in healthy volunteers and pancreatic ductal adenocarcinoma patients (PDAC) with or without obstructive jaundice (OJ) were measured by LC–MS. *GCA* glycocholic acid, *TCA* taurocholic acid, *GDCA* glycodeoxycholic acid, *TDCA* taurodeoxycholic acid, *GCDCA* glycochenodeoxycholic acid, *TCDCA* taurochenodeoxycholic acid. *N.D.* not detected. (**B**) Capan-1 cells were treated with human serum obtained from healthy volunteers and PDAC patients and the expression of vimentin was investigated by immunocytochemistry. Control (Ctrl) samples were treated with culture medium only. As a positive control gastric myofibroblasts were used. The rate of proliferation (**C**), adhesion (**D**) and viability (**E**) was measured in Capan-1 cells. a = p ≤ 0.05 vs. Control, b = p ≤ 0.05 vs. PDAC. *BAC* bile acid cocktail, *TRX* Triton-X-100.

The total serum bile acid (TSBA) concentration in healthy controls was 401.3 ± 35.38 ng/ml, whereas in PDAC + OJ patients it increased tremendously (36,055.7 ± 2182.2 ng/ml; Fig. 1A). Analysis of individual BAs has shown higher concentrations of GCA, TCA, GCDCA and TCDCA in the serum of PDAC + OJ patients. Interestingly, TCA was completely absent in healthy control, but increased dramatically in PDAC + OJ. Serum levels of TDCA were low in controls and could not be detected in PDAC + OJ patients (Fig. 1A). In PDAC patients without OJ, the TSBA concentration was 733.9 ± 118.7 ng/ml. Table 1 shows the clinicopathological characteristics and the level of BAs in human serum.

### Bile acids play a key role in the progression of PC

In the next step, we treated Capan-1 cells for 24, 48 and 72 h with serum obtained from PDAC patients (with or without OJ) and healthy volunteers (normal). Treatment with human serum induced a changed morphology and growth characteristic of the cells, therefore, we examined whether this altered morphology is associated with epithelial-mesenchymal transition (EMT). Vimentin is a structural protein that is expressed in mesenchymal cells but not in epithelial cells. In the case of PDAC + OJ a strong positive staining for vimentin was detected (Fig. 1B). In the PDAC group, only a slight staining was observed, whereas the control and the normal groups were completely negative for vimentin. These data indicate that BAs have a prominent role in the progression of cancer. To confirm this hypothesis, we supplemented PDAC serum with 0.5 mM BAs cocktail (BAC). The concentration and composition of BAC were selected on the basis of serum BAs measurements. Supplementation of PDAC serum with BAC resulted in similarly strong vimentin staining as observed for PDAC + OJ. As a positive control gastric myofibroblast were used. Moreover, we investigated proliferation, viability, and adhesion capability of the cells. As expected, serum from PDAC patients increased the rate of proliferation, adhesion and survival of Capan-1 cells compared to the normal serum (Fig. 1C–E.) Importantly, there was also a significant difference between the effect of serum from PDAC patients and that of PDAC + OJ patients, suggesting a specific role of BAs in PC pathogenesis.

### Bile acids promote proliferation, adhesion, invasion, migration and colony forming of pancreatic ductal adenocarcinoma cells

**Figure 2:**
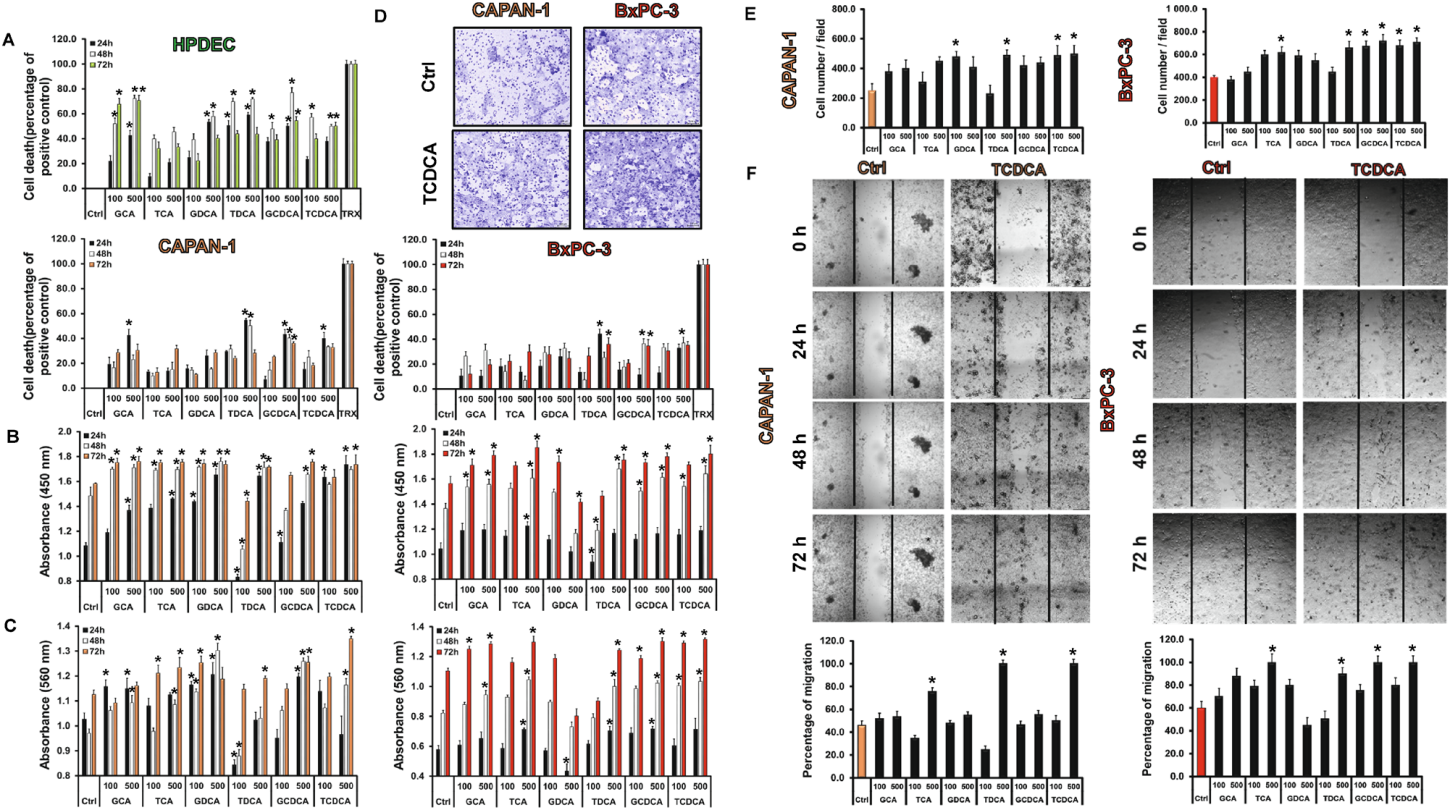
Effects of bile acid treatment on cell viability, proliferation, adhesion, invasion and migration. Capan-1 and BxPC3 cells were exposed to different concentration of bile acids (BAs) for 24, 48 and 72 h and the effects on cellular viability (**A**), proliferation (**B**), adhesion (**C**), invasion (**D**,**E**) and migration (**F**) were studied using different assays, as described in Materials and Methods. In the case of viability assay, as a positive control, 0.1% Triton-X-100 (TRX) was used. The rate of migration was investigated by the wound healing assay. Photography was taken at 0, 24, 48 and 72 h after TCDCA (500 µM) treatment using Olympus IX83 inverted microscope (Olympus cellSense Dimension software version 2.3, https://www.olympus-lifescience.com/en/software/cellsens/) and the rate of wound closure was expressed as % of migration at 72 h. Data represent mean ± SEM of three, independent experiments. * = p ≤ 0.05 vs. Control. *GCA* glycocholic acid, *TCA* taurocholic acid, *GDCA* glycodeoxycholic acid, *TDCA* taurodeoxycholic acid, *GCDCA* glycochenodeoxycholic acid, *TCDCA* taurochenodeoxycholic acid. Orange indicates Capan-1whereas red BxPC-3 cells.

The role of individual BAs in cancer progression was investigated in two PDAC cell lines. Capan-1 and BxPC-3 cells were treated with different BAs and the proliferation and metastatic potential of the cells were investigated using different cell-based assays. Interestingly, most of the BAs decreased cell viability largely in normal pancreatic cell line (HPDEC) (Fig. 2A). The average cell deaths induced by 100 µM BAs were 28.35 ± 5.78% at 24 h, 51.03 ± 4.69% at 48 h and 40.91 ± 6.2% at 72 h, whereas the effects of 500 µM BAs were more pronounced (44.32 ± 5.54% at 24 h, 62.47 ± 5.32% at 48 h and 48.86 ± 5.3% at 72 h). Low concentration (100 µM) of BAs were only slightly toxic to the Capan-1 cells; they induced cell death in approximately 16–20% of the cells. (Fig. 2A) The average cell death induced by 100 µM BAs was 16.98 ± 3.05% at 24 h, 18.82 ± 3.27% at 48 h and 20.20 ± 2.88% at 72 h. As the concentration of BAs increased (500 µM), the rate of cell death elevated depending on time. The average cell death induced by 500 µM BAs was 37.01 ± 5.91% at 24 h, 29.67 ± 5.79% at 48 h and 31.41 ± 1.19% at 72 h. Similar results were obtained with the BxPC-3 cell line.

**Figure 3:**
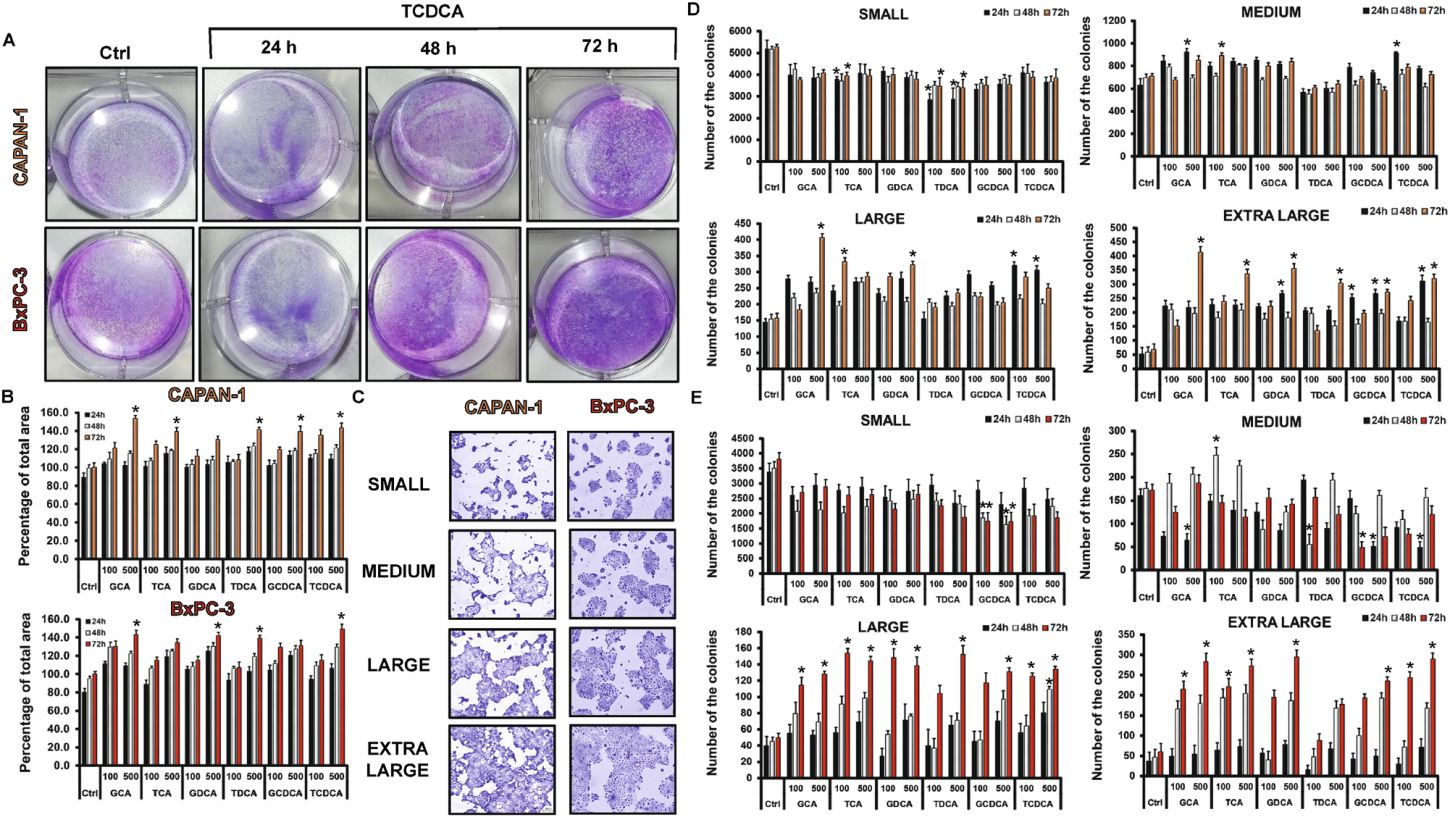
Effects of bile acid treatment on the colony forming of Capan-1 and BxPC-3 cells. Cells were exposed to different concentration of bile acids (BAs) and the colony forming ability of the cells was investigated by the clonogenic assay. (**A**) Representative pictures show the effect of taurochenodeoxycholic acid (TCDCA; 500 μM) at 24, 48 and 72 h. (**B**) Quantification of the colonies was performed using an Olympus IX83 microscope-based screening platform (Olympus cellSense Dimension software version 2.3, https://www.olympus-lifescience.com/en/software/cellsens/). (**C–E**) For the classification and counting of the colonies an automatic Olympus Cellsense Dimension software was used. (**C**) Representative images showing colonies for each cell line. Summary diagrams for Capan-1 (**D**) and BxPC-3 (**E**) cells. Data represent mean ± SEM of three, independent experiments. * = p ≤ 0.05 vs. Control. *GCA* glycocholic acid, *TCA* taurocholic acid, *GDCA* glycodeoxycholic acid, TDCA: taurodeoxycholic acid, *GCDCA* glycochenodeoxycholic acid, *TCDCA* taurochenodeoxycholic acid.

Incubation of Capan-1 and BxPC-3 cells with BAs, increased the rate of proliferation almost in all treated groups (Fig. 2B). Among BAs, the effect of TDCA was dose-dependent especially at 24 h; it suppressed proliferation of the cells (0.83 ± 0.06) at a low concentration (100 µM), and increased it (1.64 ± 0.02) at a high concentration (500 µM), depending on time. Binding of cells to extracellular matrix plays an important role in survival of cells and determines the progression and outcome of PC. Subsequently, we have investigated the effect of BAs treatment on the adhesion of Capan-1 and BxPC-3 cells to collagen 1. As shown in Fig. 2C, adhesion of cells increased with the incubation time, mainly at high doses of TCDCA-treated group. BAs treatment also promoted the invasion of Capan-1 and BxPC-3 cells, as demonstrated on Fig. 2D,E. We have also investigated the metastatic potential of cancer cells using the wound healing assay. Treatment with BAs, especially high concentration of TCDCA (500 µM), significantly increased the migration rate of both Capan-1 and BxPC-3 cells (Fig. 2F). Next, we have investigated the ability of Capan-1 and BxPC-3 cells to form colonies using the clonogenic assay. Figure 3A shows a representative picture regarding the effect of TCDCA at high (500 µM) concentration. These pictures and the summary bar chart (Fig. 3B) clearly show that high concentration of BAs increase the colony forming ability of the cells especially at 72 h. We have also investigated the size of the colony in differently treated groups (Fig. 3C–E). Furthermore, we have distinguished the colonies according to the following criteria: small: 1000–10,000 µm^2^, medium: 10,000–20,000 µm^2^, large: 20,000–30,000 µm^2^ and extra-large: 30,000–100,000 µm^2^. In the case of small colonies, a number of colonies were significantly higher in the non-treated, control group, compared to the BA-treated groups. Medium-sized colonies did not show any difference between the BA-treated and control groups. In contrast, BA treatment significantly increased the number of colonies in the large and extra-large groups, indicating that BAs induce the formation of large and extra-large colonies both in the Capan-1 (Fig. 3D) and BxPC-3 (Fig. 3E) cells, an action that promotes larger tumour tissue development.

### Expression of mucin genes in pancreatic ductal cell lines

Our results clearly demonstrate that BAs accelerate tumour processes; thus, we aimed to identify the mechanism that mediates the effects of BAs. Mucins are glycoproteins whose significance has been identified in many cancer types. To examine whether BAs are acting through the altered mucin expression, we investigated the effect of BAs on mucin expression. First, we studied the mRNA expression of mucin genes in HPDEC, Capan-1 and BxPC-3 cells using RT-PCR and TaqMan primer–probe sets, specific for mucin genes (Suppl. Table S1). We have investigated those genes (MUC1, -2, -4, -5AC, -5B, -12, -13, -15 -17, -19 and -20), which are proved to play a central role in gastrointestinal tumours, and TaqMan probe sets were available for them. In the normal cell line, the presence of MUC1, -2, -17 and -20 was shown (Table 3). In the case of Capan-1, expressions of MUC1, -4, -5AC, -5B, -13, -17 and -20 were observed, whereas in the BxPC-3 cells the presence of MUC1, -2, -4, -5AC, -5B and -13 was detected. Mucin expressions were also tested in two other PDAC cell lines, PANC-1 and MIAPaCa-2. Interestingly, much less mucin genes were detected in these cell lines (Table 3). The mucin genes used as a biomarker in PC, such as MUC4, -5AC and -5B, are expressed only in Capan-1 and BxPC-3 cells. The expression of mucin genes is summarised in Table 3.

**Table 3:** mRNA expression of mucin genes in the different pancreatic ductal cell lines.

Isoforms	Capan-1	BxPC-3	MiaPaca-2	Panc-1	HPDEC
MUC1	✓	✓	✓	✓	✓
MUC2	–	✓	–	✓	✓
MUC4	✓	✓	–	–	–
MUC5AC	✓	✓	–	–	–
MUC5B	✓	✓	–	–	–
MUC12	–	–	✓	✓	–
MUC13	✓	✓	–	–	–
MUC15	–	–	–	–	–
MUC17	✓	–	–	–	✓
MUC19	–	–	–	–	–
MUC20	✓	–	✓	–	✓

### Effect of bile acids on mucin expression

**Figure 4:**
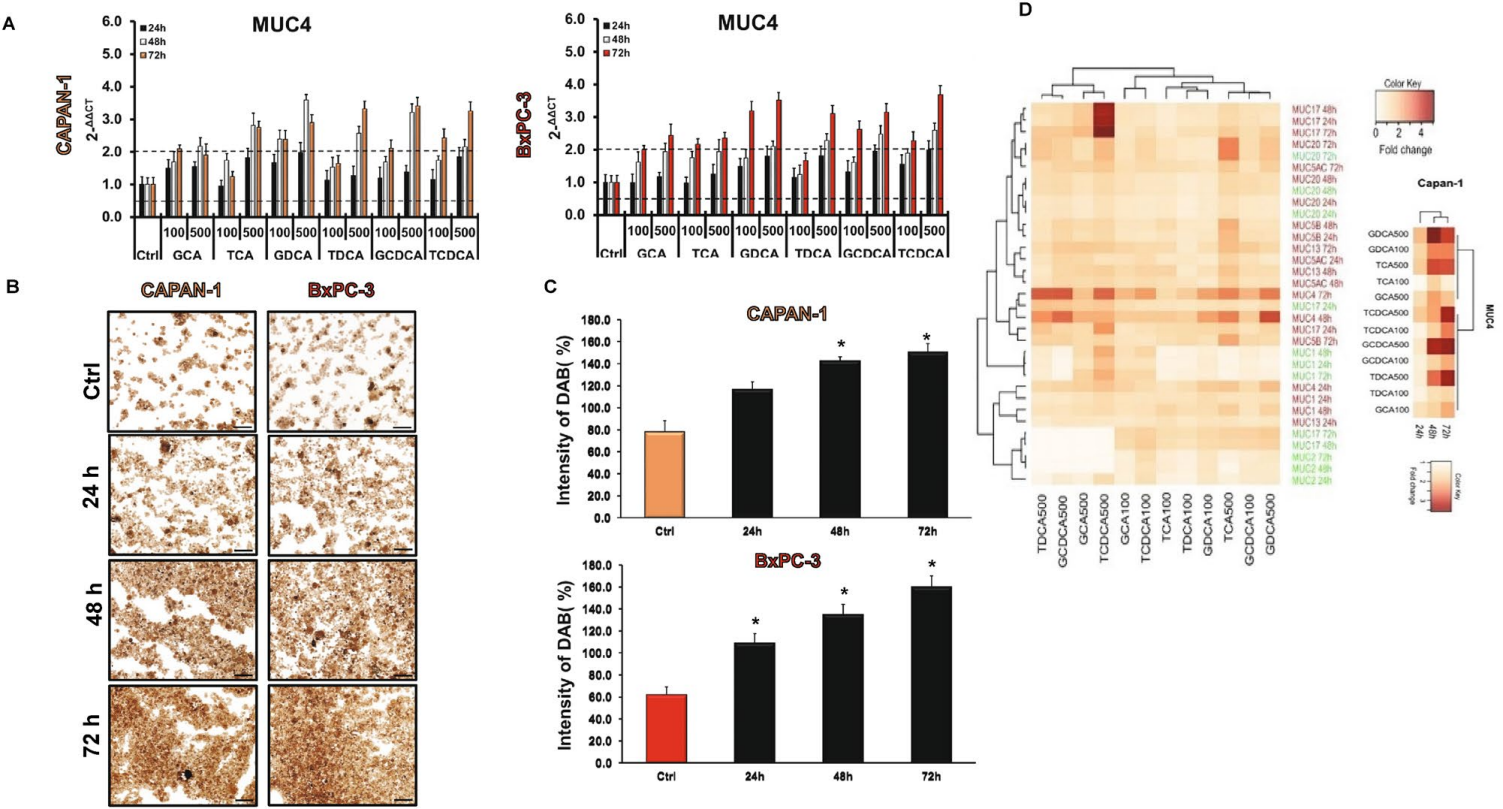
Effects of bile acid treatment on the mRNA and protein expression of mucins in pancreatic ductal cells. (**A**) Capan-1 and BxPC-3 cells were treated with different bile acids (BAs) for 24, 48 and 72 h and the relative gene expressions of MUC4 gene was investigated by real-time PCR. (**B**) Representative immunofluorescence staining of Capan-1 and BxPC-3 cells shows the expression of MUC4 after the treatment with taurochenodeoxycholic acid (TCDCA; 500 μM) for 24, 48 and 72 h. (**C**) Quantification of MUC4 protein expression. Specimens were scanned using Olympus IX83 based system (Olympus cellSense Dimension software version 2.3, https://www.olympus-lifescience.com/en/software/cellsens/) and DAB staining intensities were analysed by ImageJ software. Data represent mean ± SEM of three, independent experiments. * = p ≤ 0.05 vs. Control. Scale bar represents 100 µm. (**D**) The cluster analysis and dendrogram show the difference between the effect of BAs treatment at different concentrations and time points. Red and white colours indicate high and low expression, respectively. (Values represent the fold change in the gene expression level of MUC genes). Data represent mean ± SEM of three, independent experiments. *GCA* glycocholic acid, *TCA* taurocholic acid, *GDCA* glycodeoxycholic acid, *TDCA* taurodeoxycholic acid, *GCDCA* glycochenodeoxycholic acid, *TCDCA* taurochenodeoxycholic acid.

In the next step, we pre-treated the cells with various BAs (100 and 500 µM) for 24, 48 and 72 h and the mRNA expression of mucin genes was investigated by RT-PCR. In the normal cell line, long time incubation with the BAs decreased the expression of MUC1 and -2 in most of the treated groups (Suppl. Fig. S1A). In contrast, all of the investigated BAs dose-dependently increased the expression of MUC20 (Suppl. Fig. S1A). Treatment with BAs did not affect the expression of the other genes (data not shown). In the Capan-1 cell line, BAs treatment dose- and time-dependently upregulated the expression of MUC4 (Fig. 4A). Among the BAs, the highest effect has the conjugated forms of DC and CDC acids. In contrast, GCA and TCA induced significant increase only at higher concentrations. Interestingly, TCDCA induced a robust increase (approx. fivefold compared to the control) in the expression of MUC17 at a high concentration (500 µM), at all three incubation times (Supp. Fig. S1B). The expression of the other genes did not change significantly in most of the groups (Supp. Fig. S1B). Similarly to the Capan-1 cells, BAs treatment increased the expression of MUC4 in the BxPC-3 cell line (Fig. 4A), however it did not or hardly affect the expression of the other genes (Suppl. Fig. S1C). MUC4 has been shown to be aberrantly expressed in PC; it promotes metastasis, and it is used as a prognostic factor; thus, we investigated the expression of this gene also at a protein level. Using immunostaining, we have shown that, similar to the RT-PCR data, pre-treatment with BAs time- and dose-dependently increased the protein expression of MUC4 in both PDAC cell lines (Fig. 4B,C).

Hierarchical clustering of genes showed that TDCA, TCDCA, GCDCA and GCA (in high concentration) initiated similar MUC gene expression level changes in both cell lines and formed a separated cluster from the other BAs. The expression pattern of MUC2, -4 and -17 has changed more pronouncedly than the other genes upon BAs treatments, which suggest that these genes are more sensitive to BAs. Deeper analysis focusing on just the Capan-1 cell line showed that MUC4 pattern changed only after 48 h of the BAs treatment (Fig. 4D).

### Expression of MUC4 in human pancreatic samples

**Figure 5:**
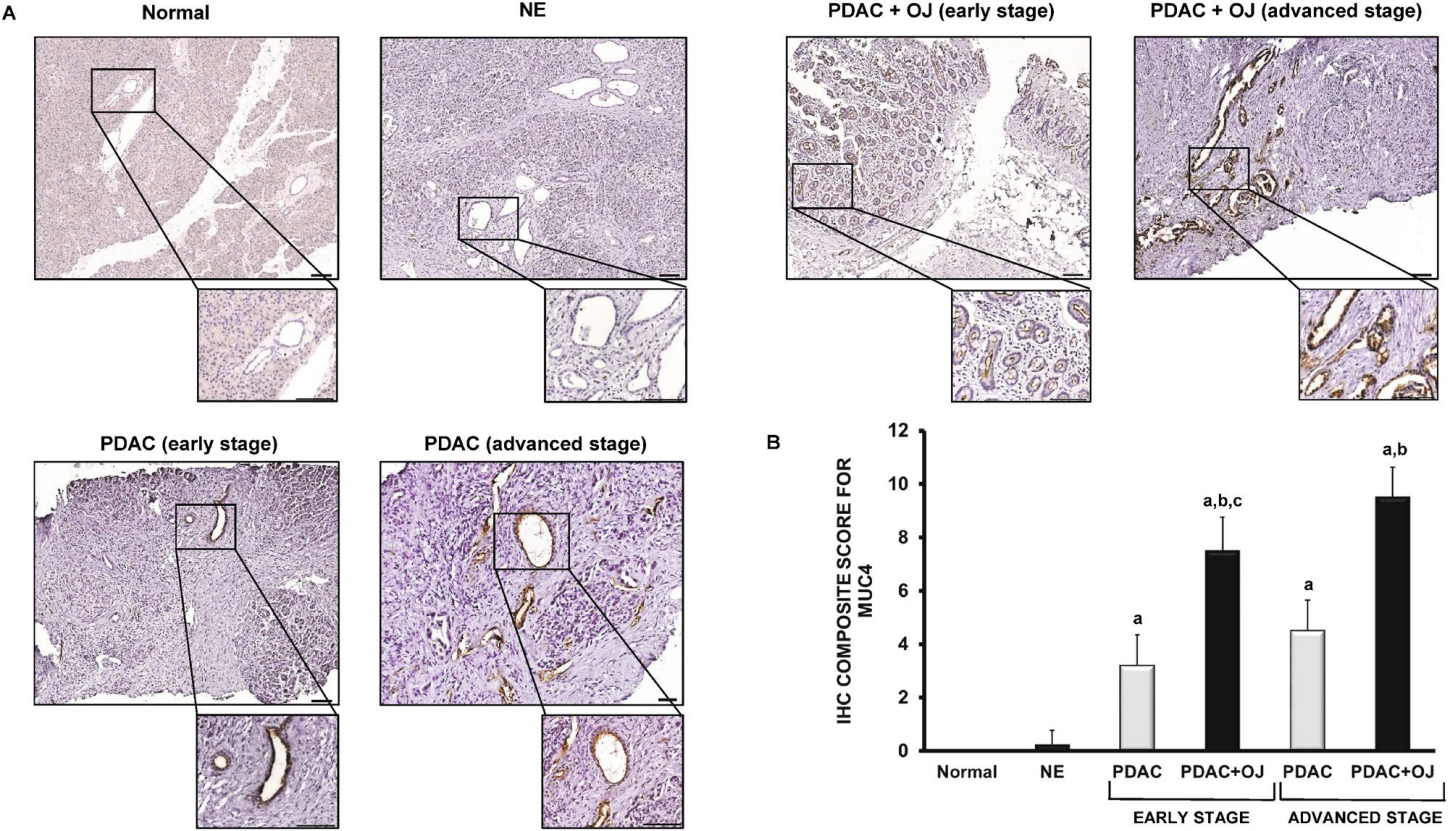
Expression of MUC4 in human pancreatic samples. (**A**) Representative immunohistochemical stainings show the presence of MUC4 in human pancreatic samples. (**B**) Composite scores of human pancreatic samples stained with anti-MUC4 antibody. Data represent mean ± SEM of 23–25 specimens/4–25 patients each group. a = p ≤ 0.05 vs. normal, b = p ≤ 0.05 vs. PDAC, c = p ≤ 0.05 vs. advanced stage. Scale bar represents 100 µm. *PDAC* pancreatic ductal adenocarcinoma; *OJ* obstructive jaundice; *NE* neuroendocrine tumour.

**Figure 6:**
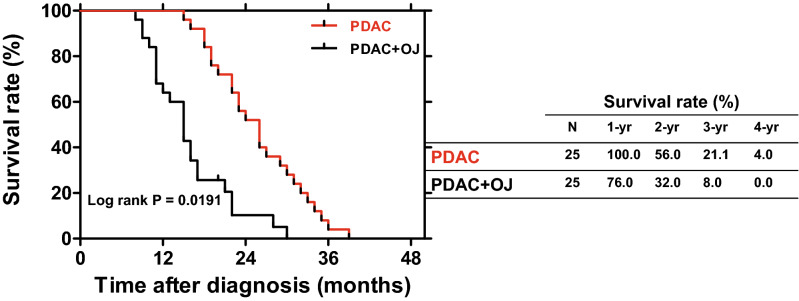
Survival curves of PDAC patients. The 4-year overall survival rate was significantly poorer in the PDAC + OJ group than in the PDAC group (log rank p = 0.0191). *PDAC* pancreatic ductal adenocarcinoma, *OJ* obstructive jaundice.

The presence of MUC4 has also been investigated in human pancreatic samples by IHC. In the normal pancreas and in NE, there was no detectable staining for MUC4 (Fig. 5A). In contrast, in the case of PDAC (with or without OJ), we observed a strong expression of MUC4 in the intra- and interlobular ducts. Interestingly, in those patients where PDAC was diagnosed without OJ, the expression of MUC4 was significantly low compared to the PDAC + OJ group. (Fig. 5B) There was no significant difference in gender, age, location of primary tumour, histological type, stage, lymphatic invasion or metastasis between the PDAC and PDAC + OJ groups (Table 2). In addition, in the PDAC + OJ group the expression of MUC4 increased with the progression of the disease, whereas in the PDAC group, there was no difference in the expression of MUC4 between the early and advanced stages. Quantification of the staining has been shown in Fig. 5B. We also examined how high serum levels of bile affects the outcome of PC. The 4-year overall survival rate of the PDAC + OJ group was significantly lower than that of the PDAC group (p = 0.0191) (Fig. 6).

### Knockdown of MUC4 decreases the carcinogenic effect of BAs

**Figure 7:**
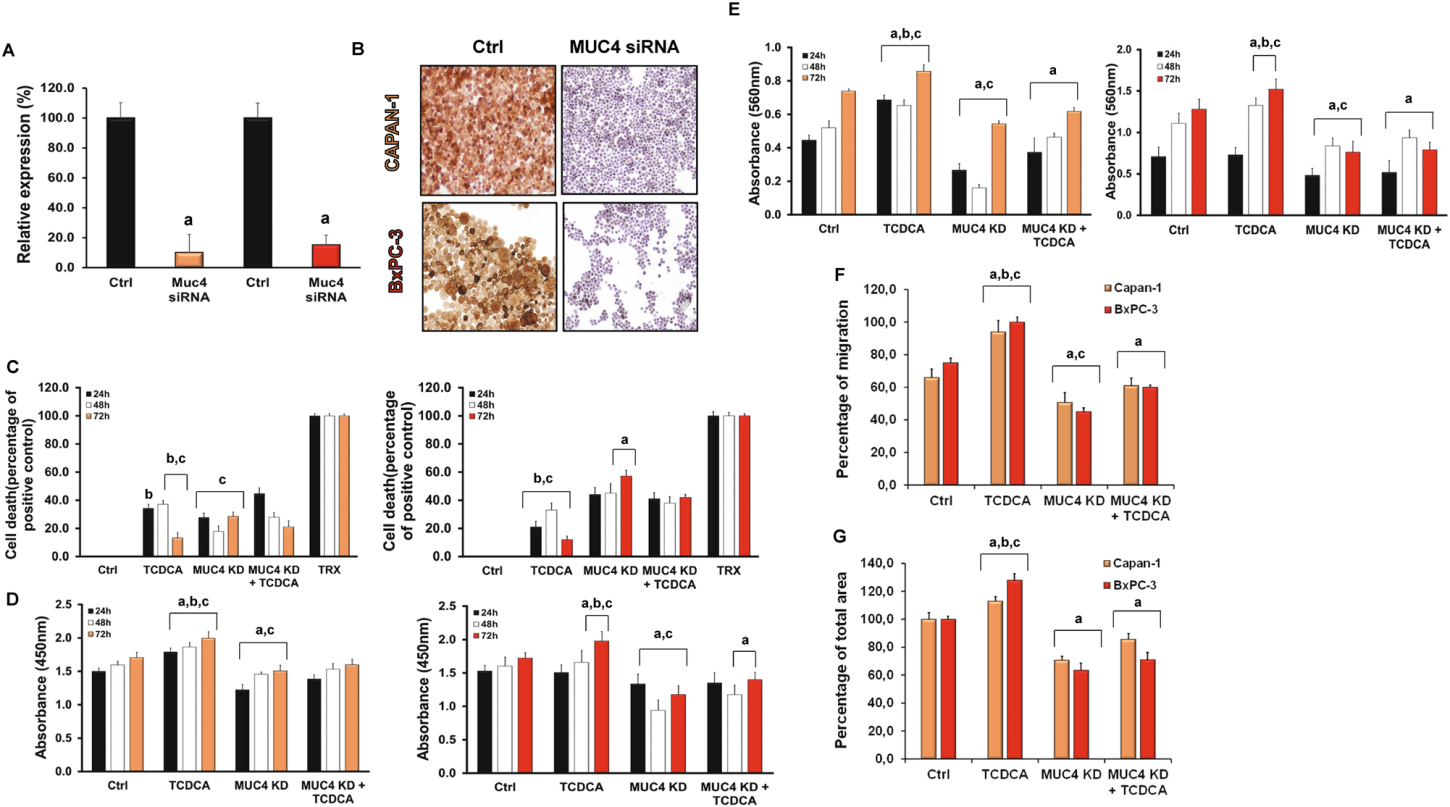
Knockdown of MUC4 in Capan-1 cells. The expression levels of MUC4 was investigated by RT-PCR (**A**) and immunohistochemistry (**B**) in control cells and in cells treated with specific siRNA for MUC4. Orange indicates Capan-1 whereas red BcPC-3 cells. The rate of viability (**C**), proliferation (**D**) and adhesion (**E**) was determined at 24, 48 and 72 h, whereas migration (**F**) and colony forming (**G**) at 72 h. Data represent mean ± SEM of three, independent experiments. a = p ≤ 0.05 vs. Control, b = p ≤ 0.05 vs. MUC4 KD, c = p ≤ 0.05 vs. MUC4 KD + TCDCA. TCDCA: taurochenodeoxycholic acid, KD: knock down.

Next, we have investigated the effect of MUC4 knockdown on the proliferation of Capan-1 and BxPC-3 cells. MUC4 was silenced by MUC4-specific siRNA. The efficiency of MUC4 knockdown was confirmed by RT-PCR (Fig. 7A) and ICC (Fig. 7B). We found that knockdown of MUC4 significantly increased cell death and decreased the rate of proliferation, adhesion, migration and colony forming in a time-dependent manner. (Fig. 7C-G) These results indicate that MUC4 is key mucoprotein in the growth of PDAC cells. In the next step we tested the effect of BAs on the MUC4-silenced cells. Among BAs, the effect of TCDCA was investigated, as this BA showed the greatest effect on both Capan-1 and BxPC-3 cells. When TCDCA was added in the absence of MUC4, an increase in the above-mentioned parameters has been observed, although it was still significantly lower than in the presence of MUC4, indicating that the effect of BAs is mediated by MUC4, although other factors also play a role in it (Fig. 7C-G and Suppl. Fig. S2.).

## Discussion

Since most of the PCs develop in the head of the pancreas, PDAC is frequently associated with increased levels of BAs in the serum; however, the effect of bile on PC progression has not been evaluated yet. In this study, we used two PDAC cell lines to show that BAs promote carcinogenic processes in which expression of MUC4 plays a huge role.

We have shown that the serum levels of BAs extremely increase in PDAC + OJ patients and the most abundant BAs are GCA, TCA, GCDCA and TCDCA. In order to investigate how elevated serum bile influences carcinogenic processes, a PDAC cell line, Capan-1 was treated with serum obtained from PDAC patients. Capan-1 is one of the most aggressive commercially available cell line; therefore, it proved to be a good model for the characterisation of PC progression^30^. High concentration of bile in the serum enhanced the tumorigenic potential of Capan-1 cells and also promoted EMT, indicating that BAs play a prominent role in the pathomechanism of PC. Previous studies indicated that the structure (number of –OH groups or the conjugation with glycine or taurine) of individual BAs determines their carcinogenic effect^31^. Moreover, the studies show that hydrophobic bile acids are mostly toxic to cells, by generating oxidative stress and DNA damage, while hydrophilic bile acids play a protective role^32^. In this study, we focused on those BAs that we detected in the serum of PDAC patients and literature data also confirm their altered concentrations in both the serum and pancreatic juice of PC patients^12,15,16^. In terms of cell survival, the normal and PC cells reacted differently to BAs. In normal cells, the higher rate of cell death was observed, especially after 48 h of BAs treatment, which indicates that, under normal conditions, the ductal cells respond by cell death to this noxious agent. Similar results have been shown on isolated, guinea pig pancreatic ducts, where the treatment of ducts with high dose (1 mM) of CDCA damaged the mitochondria and induced apoptosis in the ductal cells^32^. The apoptotic effect of BAs on normal epithelial cells has also been demonstrated in hepatocytes and in oesophageal and nasopharyngeal epithelial cells^33–35^. We hypothesised that the bile-induced cell death in the normal cells is an anti-cancer defence, by which the malignant transformation of the cells can be avoided. In contrast, cancer cells were more resistant to BAs treatment. Long-term incubation of Capan-1 and BxPC-3 cells with BAs increased their survival, which was consistent with the increased proliferation rate of these cells. The different response of normal and PDAC cells to BAs treatment can be explained by the fact that BAs are more likely to induce DNA damage than apoptosis in cancerous cells. Since gene mutations are more frequent in the damaged DNA, this favours the tumour progression^36^. In contrast, some studies have found that BAs treatment inhibit the proliferation of pancreatic cancer cell lines (PANC-1 and MIAPaCa-2) due to the cytotoxic effects of BAs^13,14^. In these studies, relatively low concentrations (< 50 µM) of BAs were used and that might cause the difference. This is also proved by the fact that, among the BAs we investigated, the effect of TDCA was dose-dependent. High concentration of this BA promoted proliferation, and low concentration strongly inhibited it. The dose-dependent effect of the unconjugated form of TDCA has also been shown on colonocyte’s^37^ and in gastric and oesophageal carcinoma^38,39^; however, the exact explanation is unknown. In addition, we have found that the adhesion, invasion, migration and colony forming ability of Capan-1 and BxPC-3 cells increased due to the BAs treatment, indicating that BAs enhance both the migratory and cell growth potential of PDAC cells. In the following, we wanted to identify the mechanism by which BAs exert their effects. Mucins can be found throughout the whole body, where they provide the hydration and lubrication of the mucosal surfaces and their pivotal role in different cancer types is generally known^40^. Depending on the tissue type, some of the genes act as a tumour suppressor and some of them promote tumour development or growth^16,18–20,41^. MUC4 is a transmembrane mucin, which has an outstanding role in PC. The expression of this gene dramatically increases in PC and the overexpression of MUC4 is associated with poor prognosis^18,41,42^. We used RT-PCR to show the presence of MUC4 in Capan-1 and BxPC-3 cells, but not in the normal cell line or in the other two PDAC cell lines. Besides MUC4, expression of MUC1, -5AC, -5B, -13, -17 and -20 have been shown in Capan-1 and the expression of MUC1, -2, -17 and -20 in the normal cells. The different expression pattern of mucins in normal and PDAC probably plays important role in cancer development. A tumour suppressor role of MUC2 has been shown in pancreatic neoplasia^43^, whereas overexpression of MUC1 and -20 correlated with poor survival in PDAC patients^43,44^. MUC5AC, -5B and -13 are absent in normal pancreas, but can be detected in pancreatic intraepithelial neoplasia and PDAC^45^. The role of MUC17 is controversial. Some data indicate that MUC17 decreases the tumorigenic potential of PDAC cells^46^, whereas others have found that this gene is aberrantly expressed in PC^47,48^.

In the normal cell line, BAs treatment decreased the expression of MUC2, and upregulated MUC20. Since MUC2 is a tumour suppressor, whereas overexpression of MUC20 favours tumour progression, these data indicate that BAs facilitate tumour development under normal conditions, by altering the expression of these mucins. In contrast, the expression of other, oncogenic mucins, such as MUC4, did not change due to the BAs treatment. In the Capan-1 and BxPC3 cell lines, BAs induced changes in the expression of MUC4 and at least two days of BAs treatment were needed to detect changes in its expression pattern. The expression of MUC17 was only affected by high concentration of TCDCA in the Capan-1 cells and it could be detected 24 h after the treatment. Using human pancreatic samples, we showed that MUC4 was completely abolished from the normal pancreatic tissue and also in NE. In contrast, strong expression was detected in PDAC, which further increased in PDAC + OJ. To exclude that elevated MUC4 levels can be explained by the more advanced stages of PDAC + OJ patients, we compared MUC4 expressions both at the early and late stages of PC. Expression of MUC4 increased with the disease progression in the PDAC + OJ group, but not in the PDAC group, indicating that the elevated level of MUC4 is due to the specific action of BAs. We also found that the presence of biliary obstruction was related to poor survival of the PDAC + OJ patients. Several studies have revealed that overexpression of MUC4 is associated with a poor clinical outcome and this gene has been reported to be an independent prognostic factor in PC^49–52^. In order to clarify the role of MUC4 in the bile-induced cancer progression, we down-regulated MUC4 by siRNA transfection and found that MUC4 act as an oncogenic mucin. The oncogenic potential of MUC4 is not surprising since silencing of MUC4 decreases the proliferation of many cancer cells. Li et al. have shown that 96 h after the transfection with shRNA, lentivector for MUC4 decreased the cell growth of BxPC-3 cells, both under in vitro and in vivo conditions^42^. Similar results have been found in other pancreatic cancer cell lines^18,41,53,54^. We have also demonstrated that inhibition of MUC4 expression significantly decreased the effect of TCDCA, one of the most effective BAs, indicating that the tumorigenic effect of bile is mediated by MUC4.

**Figure 8:**
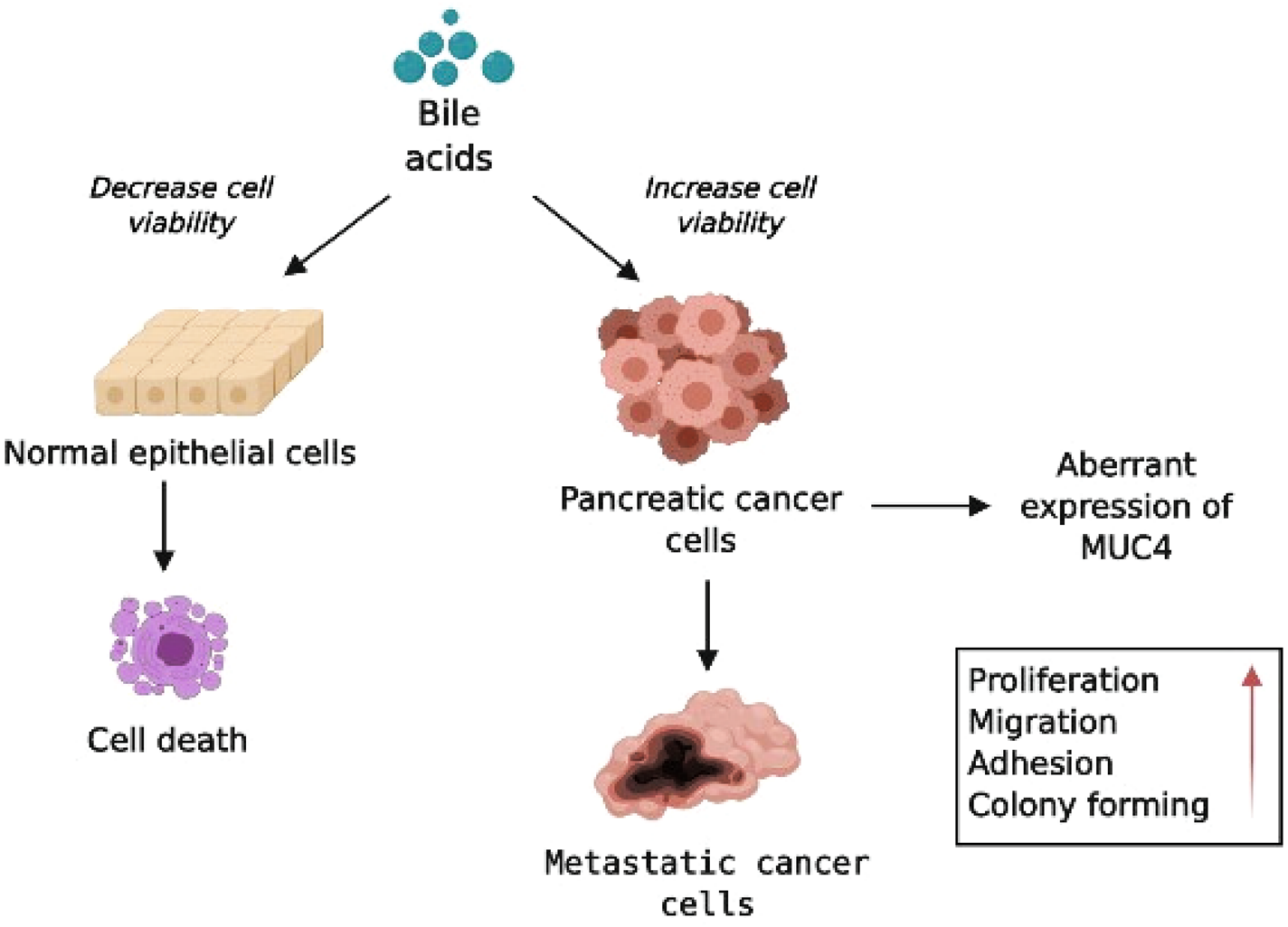
Schematic diagram of the effect of bile acids. Bile acids (BAs) decrease the cell viability of normal pancreatic ductal cells and induce cell death in order to avoid malignant transformation. In the case of pancreatic ductal adenocarcinoma (PDAC) bile promotes the tumorigenic potential of the cancer cells in which the increased expression of MUC4 plays essential role.

Figure 8 shows a hypothetic schematic figure regarding the role of BAs in PC progression. BAs induce cell death in normal pancreatic ductal cells; that are probably an anti-cancer, defensive mechanism. In contrast, elevated serum BAs levels increase MUC4 expression in PC, that presumably accelerates tumour progression. Based on these results, we believe that in PC patients with OJ, treatment of biliary obstruction needs to be done as early as possible to decrease the tumorigenic potential of PC cells and improve the life expectancy.

## Supplementary Information

Supplementary Figure 1.

Supplementary Figure 2.

Supplementary Table 1.

Supplementary Legends.

## Abbreviations

BAs: Bile acids
BAC: Bile acid cocktail
GCA: Glycocholic acid
GCDCA: Glycochenodeoxycholic acid
GDCA: Glycodeoxycholic acid
LDH: Lactate dehydrogenase
MTT: 3-(4,5-Dimethylthianol-2-yl)-2,5-diphenyltetrazolium bromide
NE: Neuroendocrine tumor
OJ: Obstructive jaundice
PC: Pancreatic cancer
PDAC: Pancreatic ductal adenocarcinoma
TCA: Taurocholic acid
TCDCA: Taurochenodeoxycholic acid
TDCA: Taurodeoxycholic acid
TSBA: Total serum bile acid
