# Supplementary figures and images for: Bile accelerates carcinogenic processes in pancreatic ductal adenocarcinoma cells through the overexpression of MUC4
Source: Sci Rep. 2020 Dec 16;10:22088. doi: 10.1038/s41598-020-79181-6 (PMC7744548; doi:10.1038/s41598-020-79181-6)

Suppl. Figure 1

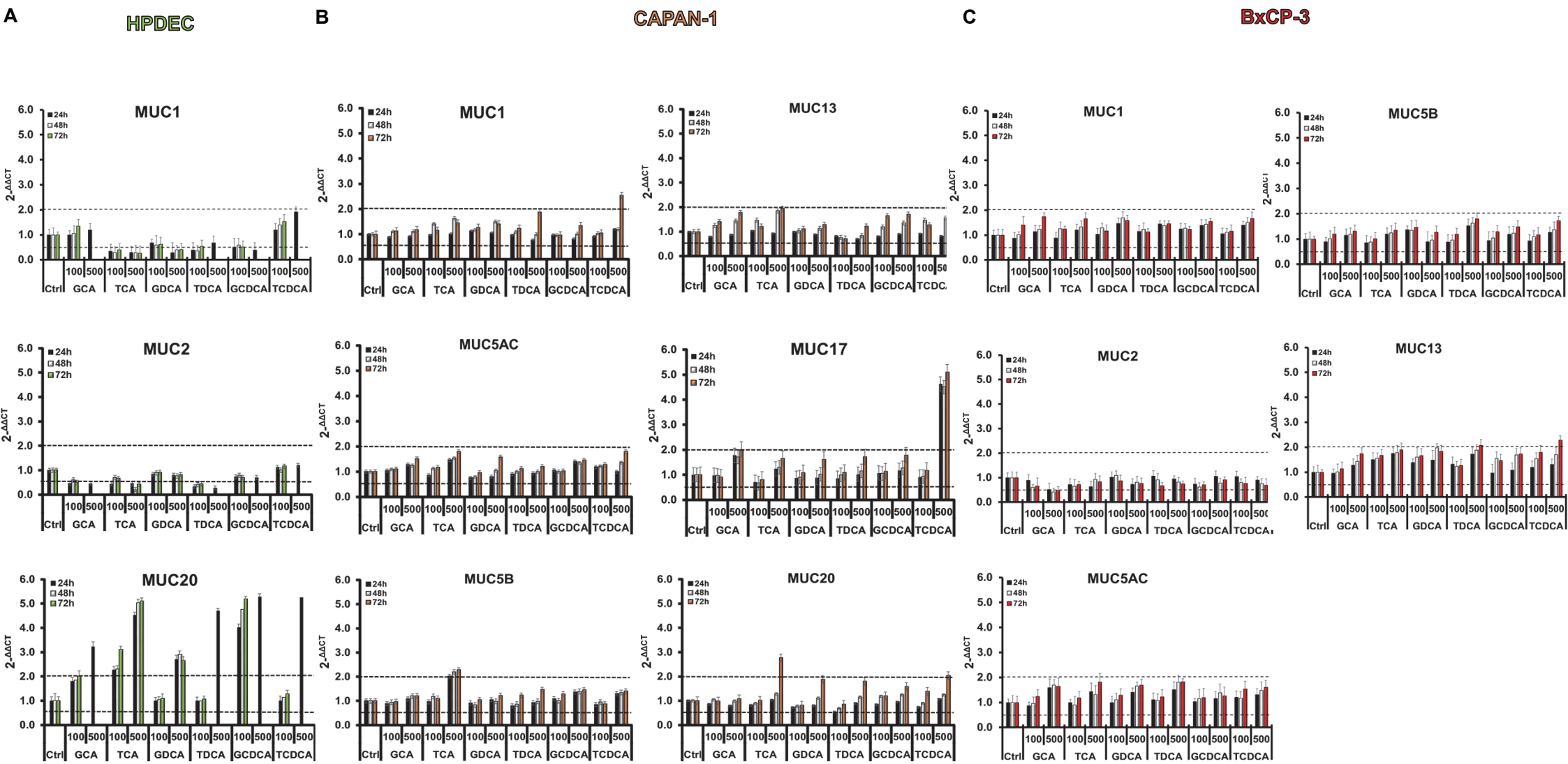

Supplement: Supplementary file 1 — Supplementary Figure 1. [file 41598_2020_79181_MOESM1_ESM.pdf]

Suppl. Figure 2

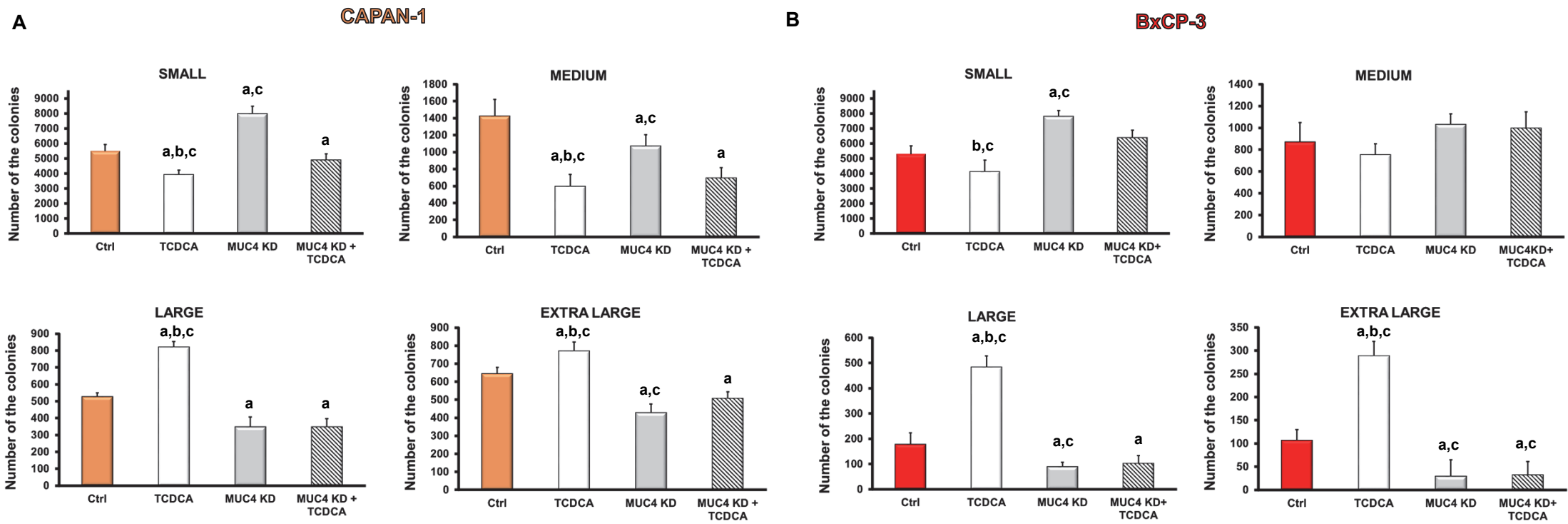

Supplement: Supplementary file 2 — Supplementary Figure 2. [file 41598_2020_79181_MOESM2_ESM.pdf]
