# Supplementary material for: Bile accelerates carcinogenic processes in pancreatic ductal adenocarcinoma cells through the overexpression of MUC4
Source: Sci Rep. 2020 Dec 16;10:22088. doi: 10.1038/s41598-020-79181-6 (PMC7744548; doi:10.1038/s41598-020-79181-6)
Supplement: Supplementary file 3 — Supplementary Table 1. [file 41598_2020_79181_MOESM3_ESM.pdf]

Suppl Table 1

Definition	Gene sym.l	REf. Seq. Acc. number	ABI TaqMan assay ID
**Homo sapiens mucin 1**	MUC1	NM_001018016.2	HS00159357.m1
**Homo sapiens mucin 2**	MUC2	NM_002457.3	HS00894052.g1
**Homo sapiens mucin 4**	MUC4	NM_001322468.1	HS00366414.m1
**Homo sapiens mucin 5AC**	MUC5AC	NM_001304359.1	HS01365616.m1
**Homo sapiens mucin 5B**	MUC5B	NM_002458.2	HS00861595.m1
**Homo sapiens mucin 12**	MUC12	NM_001164462.1	HS00419779.m1
**Homo sapiens mucin 13**	MUC13	NM_033049.3	HS00217230.m1
**Homo sapiens mucin 15**	MUC15	NM_001135091.1	HS04971585.m1
**Homo sapiens mucin 17**	MUC17	NM_001040105.1	HS05027139.s1
**Homo sapiens mucin 19**	MUC19	NM_173600.2	HS00417048.m1
**Homo sapiens mucin 20**	MUC20	NM_001282506.1	HS00380658.m1
