# Supplementary material for: Bile accelerates carcinogenic processes in pancreatic ductal adenocarcinoma cells through the overexpression of MUC4
Source: Sci Rep. 2020 Dec 16;10:22088. doi: 10.1038/s41598-020-79181-6 (PMC7744548; doi:10.1038/s41598-020-79181-6)
Supplement: Supplementary file 4 — Supplementary Legends. [file 41598_2020_79181_MOESM4_ESM.docx]

**Supplementary figure 1.** **Effect of bile acids on mucin gene expression.** HPDEC, Capan-1 and BxPC-3 cells were treated with different bile acids (BAs) for 24, 48 and 72 h and the relative gene expressions of mucin genes were investigated by real-time PCR. GCA: glycocholic acid, TCA: taurocholic acid, GDCA: glycodeoxycholic acid, TDCA: taurodeoxycholic acid, GCDCA: glycochenodeoxycholic acid, TCDCA: taurochenodeoxycholic acid.

**Supplementary figure 2.** **Colony forming ability of Capan-1 and BxPC-3 cells**. The colony forming ability of the MUC4-silenced cells was investigated at 72 h by the clonogenic assay. Quantification of the colonies was performed using an Olympus IX83 microscope-based screening platform (Olympus cellSense Dimension software version 2.3, <https://www.olympus-lifescience.com/en/software/cellsens/>). Data represent mean ± SEM of three, independent experiments. a=p≤0.05 vs. Control, b=p≤0.05 vs. MUC4 KD, c=p≤0.05 vs. MUC4 KD+TCDCA. TCDCA: taurochenodeoxycholic acid, KD: knock down.

**Supplementary table 1.** **TaqMan primer-probe sets, specific for mucin genes**
